# The Buffering Effect of Humanity of Care in the Relationship between Patient Satisfaction and Waiting Time: A Cross-sectional Study in an Emergency Department

**DOI:** 10.3390/ijerph17082939

**Published:** 2020-04-24

**Authors:** Sara Viotti, Claudio Giovanni Cortese, Jacopo Garlasco, Erika Rainero, Ifeoma Nneka Emelurumonye, Stefano Passi, Flavio Boraso, Maria Michela Gianino

**Affiliations:** 1 Dipartimento di Psicologia, Università degli Studi di Torino, 10124 Torino, Italy; sara.viotti@unito.it; 2 Dipartimento di Scienze della Sanità Pubblica e Pediatriche, Università degli Studi di Torino, 10126 Torino, Italy; jacopo.garlasco@unito.it (J.G.); erika.rainero@unito.it (E.R.); ifeomanneka.emelurumonye@unito.it (I.N.E.); mariola.gianino@unito.it (M.M.G.); 3 Azienda Sanitaria Locale Torino 3 (ASL TO3), Italy; spassi@aslto3.piemonte.it (S.P.); fboraso@aslto3.piemonte.it (F.B.)

**Keywords:** patient satisfaction, waiting time, humanity of care, environmental comfort, emergency department

## Abstract

This study aims to examine whether humanity of care and environmental comfort played a role in moderating the relationship between waiting time and patient satisfaction in an emergency department (ED). The study used a cross-sectional and non-randomized design. A total of 260 ED patients in two hospitals in Italy completed a self-report questionnaire. Moderated regression showed that after adjusting for control variables, waiting time was significantly and inversely associated with patient satisfaction. Humanity of care and environmental comfort showed a positive and significant association with patient satisfaction. Finally, the interaction term between waiting time and humanity of care was found to be significant, whereas the interaction effect between waiting time and environmental comfort was not significant. The conditional effect showed that when humanity of care was low, waiting time was negatively and significantly related to patient satisfaction. By contrast, when humanity of care was medium and high, the relationship between waiting time and patient satisfaction was not significant. These findings shed light on the key role of humanity of care in moderating the relationship between waiting time and patient satisfaction. The complex interrelations emerged should be carefully considered when interventions to foster patient satisfaction in an ED context are planned.

## 1. Introduction

Patient satisfaction is a concept that has received increasing attention in emergency medicine, reflecting an evolving focus on a patient-driven model. Historically, patient satisfaction literature has been extensively developed in countries in which the private health-care system is predominant (in particular, the US) and where patient satisfaction promotion tends to be seen as a central strategy to foster loyalty of the “patient-consumer” [1]. More recently, it has been shown that patient satisfaction is predictive of better treatment compliance, fewer malpractice complaints, and fewer professional liability litigations [2]. Therefore, in countries in which the public health-care system is prevalent, the benefits of accounting for patient satisfaction have been recognized [3,4].

Earlier studies, mostly focusing on patient background variables, established that patient satisfaction is influenced by sociodemographic, cultural, and disease-related characteristics. In particular, there is evidence that older patients are more satisfied than younger patients [5]. Additionally, illness acuity [6,7], gender [8,9], and race [10] were found to be predictors of patient satisfaction in several studies.

To gain knowledge useful to identify interventions to improve service quality in an emergency health-care setting, a growing number of studies in recent decades have addressed which specific aspects of a patient’s experience (e.g., efficiency, humanity of care, and environmental comfort) affect their satisfaction [11,12]. In this respect, emergency department (ED) literature has been particularly focused on waiting time, which is considered a central indicator of the quality of care [13,14,15]. Because of the highly unpredictable nature of the work, a long wait in the ED is quite common [16]. Long waiting times are one of the main reasons for complaints among ED patients [17]. Increased waiting time may considerably decrease patient satisfaction, for example, by fostering frustration and lessening sense of control [18]. Moreover, excessively long waits may strengthen anxiety and stress for patients and their family, demonstrating disorganization of the process and disrespect for waiting [19,20]. From an empirical point of view, studies have obtained contrasting results regarding the relationship between waiting time and patient satisfaction. Whereas some studies have found a strong association, there is also evidence disconfirming the significance of this relationship [11,14]. Moreover, empirical evidence tends to support the significance of the association between perceived waiting time and patient satisfaction but not the association between actual waiting time and patient satisfaction [19]. Thomson and colleagues [21], in their famous pioneering study, demonstrated that there is little correspondence between perceived and actual waiting time and suggested that these inconsistencies might be explained by the fact that the impact of (perceived) waiting time on patient satisfaction might be affected by various psychological and contextual aspects. Despite the potentially relevant practical implications of gaining this knowledge, to date, no studies have strived to empirically identify any of these aspects. However, given the complexity and the multifaceted nature of the patient experience, ignoring how various factors combine with each other might obscure the overall picture [19]. Therefore, to expand the current knowledge and understanding of whether and how these aspects of the patient experience in the ED interact with each other in affecting patient satisfaction, the aim of the present study was to examine the relationship between perceived waiting time and patient satisfaction. In particular, we investigated whether this relationship changed as a function of two aspects of the ED quality of care: humanity of care and environmental comfort.

Finally, considering that international literature on patient satisfaction in an emergency setting is currently dominated by research referring to Anglo-Saxon countries (in particular, the US), the present study has been carried out in Italy, a country in which the public health-care system is predominant and in which literature on patient satisfaction in the ED is insufficiently developed. The resulting findings may help identify proper interventions that promote patient satisfaction in the Italian health-care system. Moreover, findings from the present study may also expand current knowledge by identifying differences and similarities regarding patient satisfaction experienced in an ED setting across different cultural and national contexts.

### 1.1. Humanity of Care

Humanity of care refers to a patient-centred approach in which patients feel to be treated by staff with empathy and with consideration towards their needs [22,23]. The concept of humanity of care, implying humanization and personalization of care, also include the quality of the information provided by staffs as well as the involvement of patient in decision making processes about care. 

As demonstrated by a number of studies [13,14,22], humanity of care represents a key predictor of patient satisfaction. On the other hand, no previous studies were designed to investigate the combined effected between waiting time and humanity of care on patient satisfaction. However, the idea that the negative relationship between waiting time and patient satisfaction might by moderated by humanity of care has been indirectly suggested by various evidence. A recent qualitative study, conducted on ED patients [22], concluded that the adoption of a human approach in caring is central to foster ED patient satisfaction, existing as a factor that affects patient satisfaction not only directly, but also indirectly by influencing the effect that other aspects of the patient experience, including waiting time, have on it. In the same direction, a study [13], based on 11,352 ambulatory patients of a private medical centre system in the US, suggested that satisfaction with waiting time was affected by the quality of the interactions of patient-care providers rather than the actual waiting time. Based on this, we formulate the following hypothesis:

**Hypothesis** **(H1).** *The negative relationship between waiting time and patient satisfaction is moderated by humanity of care.*

### 1.2. Environmental Comfort

Environmental comfort is considered an indicator of the quality of the care and refers to the accessibility, cleanness, and cosiness of the facilities (e.g., waiting room) [24]. 

Evidence has demonstrated a significant association of environmental comfort with patient satisfaction, even if weaker in comparison to humanity of care [25]. A study carried out in an ED department of an Indian hospital revealed that aspects concerning humanity of care made up, on average, a higher percentage of variance of patient satisfaction, when compared with aspects describing environmental comfort (9.65% against 8.48%). While environmental comfort may not represent a key predictor of patient satisfaction in an ED context, a seminal paper [25] suggested that this factor may be important in order to understand how waiting time affects patient satisfaction, explaining how any deviation in the environment from an individual comfort standard may prolong the perception of waiting time. In view of that, and in consideration of the well-documented significant association between perceived waiting time and patient satisfaction [19], the following can be hypothesized:

**Hypothesis** **(H2).** *The negative relationship between waiting time and patient satisfaction is moderated by environmental comfort.*

## 2. Materials and Methods

### 2.1. Procedure

The study, with a cross-sectional and nonrandomized design, was conducted in the EDs of two medium-sized hospitals (i.e., Ospedale degli Infermi and Ospedale Edoardo Agnelli) in the western suburban area of Turin (Piedmont, Italy), both belonging to the Health Care Unit “ASL Torino 3”.

These hospitals, equipped with clinical and surgical units, are hubs for the Emergency Department of Health Care Unit. Rivoli Hospital has 280 beds and admits 60,000 patients to the emergency room each year. Pinerolo Hospital has 230 beds and admits 52,000 patients to the emergency room each year.

The data collection was carried out in the period May to October 2019 on multiple days of the week from Monday to Sunday. All adult patients accessing the EDs who met the following inclusion criteria were considered potentially eligible for the study:

Over 18 years on the admission day;
Individual admission to the ED via standard triage procedures;
Absence of noticeable mental impairment.

Patients who met all inclusion criteria were enrolled immediately after their discharge from the ED by a researcher. Each participant was asked to sign an informed consent form regarding the purposes of the study and the data treatment. Participants were also assured of data anonymity and confidentiality. After being provided with the questionnaire, participants were asked to complete it outside the examination room to remove all possible biases due to the presence of healthcare professionals. The research protocol was in accordance with the Helsinki Declaration (and subsequent revisions) and the Italian regulations on data protection and privacy (Law n. 196/2003). No further approval was required since the study design did not include the administration of any invasive medical treatment.

### 2.2. Measures

Major study variables were collected with a self-report questionnaire using the following items and scales.

Waiting time. Perceived waiting time was measured using a single item, adapted from the literature [13,25]: “After you had been assigned with a triage tag, how long did you wait before being visited by a physician?” Response options range from 1 to 7 (1 = less than 30 min, 2 = about an hour, 3 = about two hours, 4 = about three hours; 5 = about four hours, 6 = about five hours, 7 = six hours or more).

Patient satisfaction. In accordance with previous research conducted in the Italian health-care system [23], patient satisfaction was measured with a single item: “How do you rate satisfaction with the service received?” The responses were given on a 5-point scale ranging from “very low” to “very high”.

Humanity of care. Humanity of care was measured with a 9-item scale. The items were adapted from the works by Murante et al. [23], Bleustein et al. [13], and Repplinger et al. [26], and the scale aimed to assess the patients’ perceptions of staff regarding courtesy, willingness displayed to meet the needs of the patient, confidence inspired, and the acceptance of the needs for humanization and personalization of care (see Table 1).

Environmental comfort. Environmental comfort was measured using 3 items drawn from previous studies [27]. These items assessed the accessibility of the facilities, the cleanliness of the waiting room and the comfort of the waiting room (see Table 1).

Responses to items from both the humanity of care and environmental comfort scales were given on a five-point Likert scale (labelling varied, according to the wording of the items, e.g., 1 = very poor, 5 = very good; 1 = not at all, 5 = totally; 1 = totally negatively, 5 = totally positively).

Control variables. Background variables (i.e., age, gender, citizenship) and triage tags (as inpatients and outpatients) were retrieved from the administrative records of the hospital. Each background variable was extracted from the alphanumerical code assigned to each patient in the emergency room during hospital admission.

### 2.3. Statistical Analyses

Preliminary analyses (e.g., descriptive, analyses, Pearson correlations, Analysis of Variance, χ^2^ test) were conducted with SPSS 25. To ascertain the psychometric properties of two multi-item scales, preliminary analyses also included a principal component analysis (PCA, rotation: oblimin; number of factors to extract: 2).The solution obtained is reported in Table 1 (Kaiser–Meyer–Olkin for sampling adequacy = 0.83; Bartlett’s test = 1508.27, df = 66, *p* = 0.0001). The first factor extracted was humanity of care, which explained 43.93% of the variance and consisted of 9 items (factor loading included between values 0.54 and 0.84). The second factor extracted was environmental comfort, which included 3 items (factor loading included between 0.54 and 0.84) and explained 13.80% of the variance. Cronbach’s alpha further confirmed the adequacy of psychometric proprieties, with values of 0.87 and 0.81 for humanity of care and environmental comfort, respectively.

**Figure 1:**
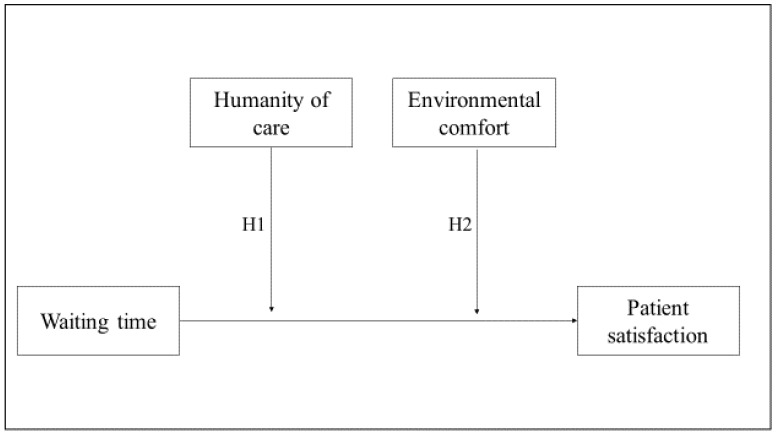
The research model.

The moderation analysis was carried out by selecting model 2 in PROCESS-Version 3 [28], which was developed by Preacher and Hayes [29] for SPSS. A representation of the research model is reported in Figure 1. In Step 1, patient satisfaction was included as the dependent variable (Y), waiting time as the independent variable (X), and environmental comfort and humanity of care as moderators. In Step 2, control variables were added as covariates. By selecting Model 2, the software automatically built and included in the model the two cross-products (interaction terms) between the independent variables and the two moderators at both steps. The independent variables included in the model were checked for multicollinearity, using tolerance and the variance inflation factor (VIF). VIF-values greater than 10 and tolerance-values smaller than 0.10 may indicate multicollinearity. There were no signs of multicollinearity in any step of the regression model since the highest value of VIF and the lowest value of tolerance, 1.628 and 0.614, respectively, was obtained by environmental comfort in Step 2.

To ascertain moderation, any significant interaction effect was further analysed by inspecting the conditional effects of the independent variables on the dependent variables at three levels of the moderator (±1 standard deviation (SD), mean (M)). The bias-corrected 95% confidence interval (CI) was calculated with 5000 bootstrapping resamples.

## 3. Results

### 3.1. Descriptive Analyses

In total, 297 patients were asked to filled out the questionnaire. As 37 patients refused to take part to the study (rejection rate, about 12.5%), the final sample consisted of 260 patients (160 from Rivoli Hospital and 100 from Pinerolo Hospital). The majority of patients were Italian (*n* = 242, 93.1%), with ages ranging between 18 and 91 years (M = 56.15, SD = 18.29). The sample was balanced from the point of view of gender, with 50.8% (*n* = 132) male and 49.2% (*n* = 128) female. At admission, the majority of respondents were coded as low-priority patients, since 88 (30.8%) of them obtained a white tag (no urgency), 171 (65.8%) received a green tag (low urgency), and only 9 (3.5%) were assigned a yellow tag (medium urgency). Similarly, when respondents were discharged, 42 (16.2%) were labelled with a white code, 209 (80.4%) with a green code, and 9 (3.5%) with a yellow code.

### 3.2. Univariate Analyses

As reported in Table 2, correlations between patient satisfaction and waiting time (r = −0.23, *p* = 0.001), humanity of care (r = 0.69, *p* = 0.001), and environmental comfort (r = 0.42, *p* = 0.001) were all significant and in the expected direction.

**Figure 2:**
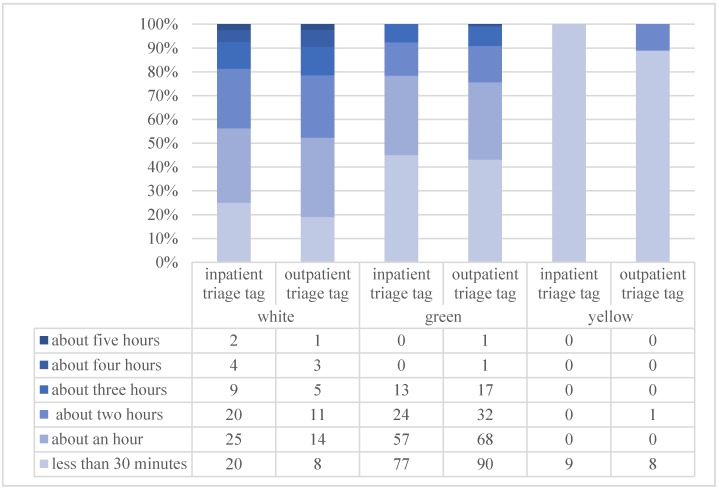
Histogram representation of the cross between waiting time (less than 30 min; about an hour; about two hours; about three hours; about four hours; about five hours) and inpatient/outpatient triage tags (white = no urgency; green = low urgency; yellow = medium urgency).

As expected, the χ^2^ test highlighted the dependency between waiting time and both inpatient (χ^2^ = 36.996, *p* = 0.0001) and outpatient (χ^2^ = 29.155, *p* = 0.001) triage tags (see Figure 2). 

Regarding control variables, patient satisfaction reported no significant associations with gender, citizenship, hospital, inpatient and outpatient triage tags. On the other hand, age was found to be positively correlated with patient satisfaction (r = 0.21, *p* = 0.001).

### 3.3. Moderated Regression Analyses

Table 3 reports the results of the moderated hierarchical regression in which patient satisfaction was entered as a dependent variable.

In the first step, when both the main effects (i.e., waiting time, humanity of care, environmental comfort) and the two interaction terms (i.e., waiting time*humanity of care and waiting time*environmental comfort) were entered, the overall variance explained by the model was 52%. By inspecting single paths, the waiting time was found to be significantly and inversely associated with patient satisfaction (B = −0.61, *p* = 0.01). Humanity of care (B = 0.06, *p* = 0.001) showed a positive and significant association with the dependent variable, but environmental comfort did not (B = 0.04, *p* = 0.06). Finally, whereas the interaction term between waiting time and humanity of care was found to be significant (B = 0.01, *p* = 0.06), the interaction effect between waiting time and environmental comfort (B = 0.01, *p* = 0.76) was not significant. In the second step, the model was adjusted for the control variables (overall variance explained: 56%). With the only exception of environmental comfort, which in the second step was found to be significantly associated with patient satisfaction (B = 0.05, *p* = 0.02), no modifications in terms of significance and direction, if compared with the previous step, were observed on the association between major study variables (both main effects and interaction terms) and patient satisfaction. Regarding control variables, no significant associations were found between gender, citizenship, impatient triage tag, and outpatient triage tag with patient satisfaction. On the other hand, age was found to be positively associated with patient satisfaction (B = 0.006, *p* = 0.02). Moreover, at Pinerolo Hospital, patient satisfaction was found to be significantly higher than that at Rivoli Hospital (B = 0.18, *p* = 0.01).

**Figure 3:**
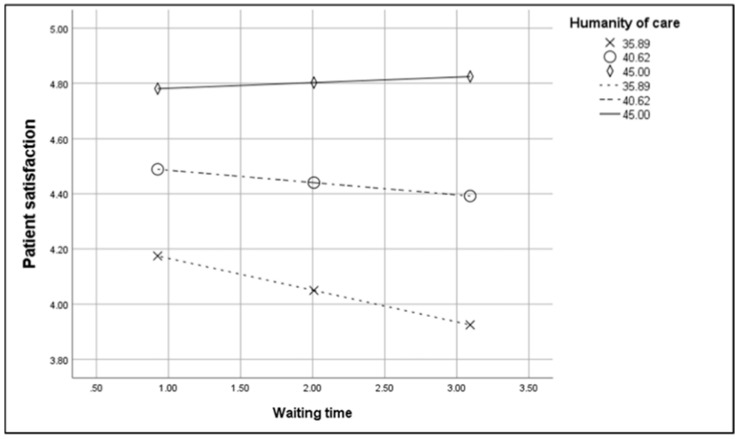
The effect of waiting time on patient satisfaction at low, medium, and high levels of humanity of care.

The interaction effect between waiting time and humanity of care is plotted in Figure 2. The conditional effect (Figure 3) showed that when humanity of care was low (−1 SD), waiting time was negatively and significantly related to patient satisfaction (B = 0.09, t = −2.33, *p* = 0.02, LLCI = −0.17, ULCI = 0.01). In contrast, when humanity of care was medium (M, B = 0.03, t = −1.24, *p* = 0.21, LLCI = −0.10, ULCI = 0.02) and high (+1 SD, B = 0.01, t = 0.27, *p* = 0.78, LLCI = −0.07, ULCI = 0.09), the relationship between waiting time and patient satisfaction was not significant. Due to the non-significant effect of the interaction term involving environmental comfort, only the conditional effect for humanity of care was analysed. 

Overall, these findings suggested to accept H1 and to rejected H2.

## 4. Discussion

The findings revealed a significant association between waiting time and patient satisfaction. However, this relationship was found to be moderated by humanity of care: only in the case of a low level of humanity of care, and not in case of medium and high level of humanity of care, the relationship between waiting time and patient satisfaction was significant (H1 confirmed). On the other hand, environmental comfort did not show a moderating role between waiting time and patient satisfaction (H2 rejected).

In the ED, it may be difficult to maintain a high standard in humanity of care because of high patient turnover; in addition, there are many conditions that may jeopardize the quality of the interaction with patients, such as noise, lack of privacy, frequent interruption, limited time unpredictability, intense work pace, and overcrowding [22,30]. This study underscores the importance of maintaining a humanistic approach in each patient interaction, despite the complexity of the emergency setting. Humanism refers to fostering an interaction in which the patients feel like a person and less like a number or a case [22,23], while also displaying interest and concern for patient well-being. The adoption of a humanistic approach in providing a service by the ED staff may help alleviate patient anxiety and negative emotions related to critical health conditions, thus contrasting the negative effect of waiting on overall patient satisfaction. A service characterized by poor humanity of care combined with long waiting time might make patients unconfident regarding the quality of care provided, thus leading to perceive wait as a waste of time. When humanity of care is low, anxiety may increase fear of health conditions, thus decreasing patient satisfaction. It is also possible to assume that this condition may also favour unsafe patient behaviours highlighted in previous studies, such as leaving the ED facilities before receiving a treatment [31,32]. On the other hand, patients might be less upset with long waits when they feel their needs considered and that ED staff show attention for their health condition. Humanity of care might have a containment function for patient anxiety. In this scenario, patients may find it reasonable to wait to receive personalized and high-quality care.

Contrary to our expectations, environmental comfort did not moderate the relationship between waiting time and patient satisfaction. Moreover, it also directly and weakly affected patient satisfaction, since in one step of the moderated regression, the association was found to be not significant. This is inconsistent with some previous studies in literature that have suggested that environmental comfort represents an important factor for patient satisfaction, as comfortable facilities (e.g., comfortable seats) may help people bear the wait [24,25]. Even if this could be true in general for waiting patients in other care services (e.g., waiting to be seen by the family doctor), our study suggests that this is not entirely applicable to an ED patient experience. Many patients, even if tagged with a low-priority code, might be worried about their health conditions and anxious about receiving a diagnosis and treatment. Due to their worries, during the wait, patients might not care about the comfort of the environment. In this view, it makes sense that environmental comfort is not capable of buffering the negative effect of waiting time on patient satisfaction. The marginal role of environmental comfort in an ED context, from a patient satisfaction perspective, has been suggested by a previous study [17], which showed that comfortable facilities are infrequent reasons for patient complaints compared to waiting, treatment, and communication.

The complex interrelations that emerged among humanity, waiting time, and patient satisfaction should be carefully considered when interventions to foster patient satisfaction in an ED context are planned. In particular, staff communication skills, emphatic attitudes, and involvement of patients in the decision-making processes have emerged as central to ensuring a positive patient experience. Therefore, in line with previous studies [14], our findings suggest that specific training courses aimed at supporting the staff (both physicians and nurses) to improve these skills might be useful to improve patient satisfaction. This type of intervention may make emergency staff more aware of the importance of developing effective and empathic communication with patients as well as of involving them, whenever possible, in the decision making process regarding diagnosis and treatment. Moreover, ensuring patient understanding of the diagnosis and treatment plan has been demonstrated to not only lead to higher patient satisfaction but also ensure overall safer care [33].

There is also a large corpus of studies that have demonstrated that negative attitudes of staff towards patients may be caused by stressful conditions in the workplace, such as work overload, poor job autonomy, and poor support from colleagues and supervisors [34]. In particular, the risk for emergency staff to incur burnout, a syndrome caused by stressful working conditions [35,36] and characterized by feeling of exhaustion, the tendency to treat patients like objects and to become indifferent and apathetic towards them, is well known [37]. In this view, to improve humanity of care, it is essential to contrast burnout by promoting healthier working conditions. Example actions in this direction might be the implementation of job analysis and job redesign programmes aimed at identifying and correcting working conditions potentially stressful (e.g., excessive workload). In addition, reflective practice groups [38] may represent a useful tool to sustain both the quality of the work process and the quality of care. A reflective practice group is a technique that provides an opportunity for a work team to collectively examine daily practice and learn through—and from—experience. It can be used with the idea of identifying shared visions regarding ways to improve the quality of care and the management of the relationship with patients.

The present study is not without limitation. The representativeness of the results may be a limiting factor. Given the use of a non-randomized sampling procedure, the research findings may not be generalizable. It is also possible that a different sample of patients may have yielded different findings.

Similar to previous research, the present study involved almost exclusively lower-acuity patients, that is, patients tagged with a low-priority code. Even if this aspect favours the comparability of our findings with literature, it is important to remember that it was widely demonstrated that illness acuity at the time of admission strongly affects patient satisfaction [6,7]. Therefore, future studies should find strategies compatible with respect to ethical standards to involve a larger number of patients tagged with higher-priority codes in order to have a more representative sample and to account for any difference in the associations among study variables across patients with different levels of illness acuity.

Finally, regarding waiting time, our study measured only perceived waiting time and not the actual waiting time. Although literature has highlighted that perceived waiting time is more strongly associated with patient satisfaction, future studies might consider both actual and perceived waiting time to better understand the relationship between perceived quality of care, patient satisfaction, and waiting time. For instance, future research may examine whether humanity of care has a role in explaining the difference between actual and perceived waiting time and whether and how this difference may affect patient satisfaction.

## 5. Conclusions

The present study is of importance since it is the first study, to the best of our knowledge, that tested the moderating role of humanity of care and environmental comfort in regards to waiting time and patient satisfaction. In particular, a key added value of this study was that we highlighted the way in which the various aspects of the care experience affected patient satisfaction in the ED context and how it is more complex than previously thought. Generally, our findings confirmed previous studies that demonstrated that waiting time and humanity of care are central to ensuring quality of care. However, those previous studies tended to assume that the humanity of care and waiting time independently influence patient satisfaction. Therefore, the present study advances the current knowledge by shedding light on the mechanism by which humanity of care buffers the negative effect of waiting time on patient satisfaction.

## Figures and Tables

**Table 1:** Principal component analysis and item content of multi-item measures.

	Item	M(ds)	Factor Loading	
			Factor 1 (Humanity of Care)	Factor 2 (Environmental Comfort)
1	Courtesy of the Triage Personnel	4.52 (0.831)	0.845	
2	Ability of Triage Personnel to Understand the Severity of the Health Problem	4.40 (0.839)	0.832	
3	Concern the Care Providers Showed for Questions or Worries	4.39 (0.757)	0.755	
4	Time Taken by Care Providers in Listening to Patients	4.50(0.738)	0.751	
5	Courtesy of the Care Providers	4.65(0.600)	0.718	
6	Trust towards Care Providers	4.70(0.544)	0.697	
7	Humanness of Care Providers (Being Treated “As a Person”)	4.66(0.711)	0.662	
8	Care Providers’ Efforts in Including Patients’ in Decision about Treatment	4.27(0.899)	0.653	
9	Clarity of Information Received from Care Providers (Physicians and Nurses)	4.50(0.683)	0.541	
10	Comfort of the Waiting Room	3.64(1.253)		0.901
11	Cleanness of the Waiting Room	3.76(1.080)		0.896
12	Accessibility to the Facilities (Parking, Direction Information)	3.35(1.232)		0.744

Note. Factor loading < 0.40 were not reported.

**Table 2:** Descriptive analysis and Pearson correlations.

Major Study Variables	M(sd)	1	2	3	4
1. Patient Satisfaction	4.42(0.73)	1			
2. Waiting Time	1.01(1.08)	−0.23 **	1		
3. Humanity of Care	40.61(4.70)	0.69 **	−0.24 **	1	
4. Environmental Comfort	10.73(3.05)	0.42 **	0.01	0.41 **	1

** *p* < 0.01.

**Table 3:** Moderated regression analysis (dependent variable: patient satisfaction).

Variable	Step 1					Step 2				
	B	se	*p*	95% CI		B	se	*p*	95% CI	
				LLCI	ULCI				LLCI	ULCI
Waiting Time	−0.61	0.24	0.012	−1.10	−0.13	−0.65	0.24	0.007	−1.13	−0.17
Humanity of Care	0.06	0.01	0.001	0.02	0.09	0.05	0.01	0.002	0.01	0.08
Environmental Comfort	0.04	0.02	0.062	−0.00	0.09	0.05	0.02	0.022	0.02	0.01
Waiting Time * Humanity of Care	0.01	0.00	0.026	0.01	0.02	0.01	0.00	0.013	0.01	0.02
Waiting Time * Environmental Comfort	0.00	0.01	0.769	−0.01	0.02	0.01	0.01	0.913	−0.01	0.02
Age						0.00	0.00	0.002	0.00	0.01
Gender (1 = male)						0.02	0.06	0.692	−0.10	0.15
Citizenship (1 = Italian)						0.06	0.13	0.621	−0.19	0.33
Impatient Triage Tag						−0.07	0.08	0.372	−0.23	0.08
Outpatient Triage Tag						0.01	0.10	0.883	−0.18	0.21
Hospital (1 = Pinerolo)						0.18	0.07	0.017	0.03	0.34
	R^2^ = 0.52 F = 53.21 *p* = 0.0001					R^2^ = 0.56 F = 26.97 *p* = 0.0001				

Note. listwise *n* = 243 (Step 1) *n* = 241(Step 2); LLCI = lower levels for confidence interval; ULCI = upper levels for confidence intervals; se = standard error; * = cross-product sign; F = Fisher test.
